# An alternative approach to reduce algorithm‐derived biases in monitoring soil organic carbon changes

**DOI:** 10.1002/ece3.5308

**Published:** 2019-05-30

**Authors:** Weixin Zhang, Yuanqi Chen, Leilei Shi, Xiaoli Wang, Yongwen Liu, Rong Mao, Xingquan Rao, Yongbiao Lin, Yuanhu Shao, Xiaobo Li, Cancan Zhao, Shengjie Liu, Shilong Piao, Weixing Zhu, Xiaoming Zou, Shenglei Fu

**Affiliations:** ^1^ Key Laboratory of Geospatial Technology for the Middle and Lower Yellow River Regions, Ministry of Education, College of Environment and Planning Henan University Kaifeng China; ^2^ Key Laboratory of Vegetation Restoration and Management of Degraded Ecosystem, South China Botanical Garden Chinese Academy of Sciences Guangzhou China; ^3^ Hunan Province Key Laboratory of Coal Resources Clean‐utilization and Mine Environment Protection Hunan University of Science and Technology Xiangtan China; ^4^ University of Chinese Academy of Sciences Beijing China; ^5^ State Key Laboratory of Plateau Ecology and Agriculture, Qinghai Academy of Animal and Veterinary Sciences Qinghai University Xining China; ^6^ College of Urban and Environmental Sciences Peking University Beijing China; ^7^ Jiangxi Provincial Key Laboratory of Silviculture, College of Forestry Jiangxi Agricultural University Nanchang China; ^8^ College of Life Sciences Henan University Kaifeng China; ^9^ Xishuangbanna Tropical Botanical Garden Chinese Academy of Sciences Mengla China; ^10^ Department of Biological Sciences Binghamton University, the State University of New York Binghamton New York; ^11^ Department of Environmental Sciences University of Puerto Rico San Juan Puerto Rico

**Keywords:** algorithm‐derived biases, basal mineral‐matter reference systems, equivalent mineral‐matter volume, reference systems, SOC comparability, soil organic carbon, soil volume change

## Abstract

Quantifying soil organic carbon (SOC) changes is a fundamental issue in ecology and sustainable agriculture. However, the algorithm‐derived biases in comparing SOC status have not been fully addressed. Although the methods based on equivalent soil mass (ESM) and mineral‐matter mass (EMMM) reduced biases of the conventional methods based on equivalent soil volume (ESV), they face challenges in ensuring both data comparability and accuracy of SOC estimation due to unequal basis for comparison and using unconserved reference systems. We introduce the basal mineral‐matter reference systems (soils at time zero with natural porosity but no organic matter) and develop an approach based on equivalent mineral‐matter volume (EMMV). To show the temporal bias, SOC change rates were recalculated with the ESV method and modified methods that referenced to soils at time t1 (ESM, EMMM, and EMMV‐t1) or referenced to soils at time zero (EMMV‐t0) using two datasets with contrasting SOC status. To show the spatial bias, the ESV‐ and EMMV‐t0‐derived SOC stocks were compared using datasets from six sites across biomes. We found that, in the relatively C‐rich forests, SOC accumulation rates derived from the modified methods that referenced to t1 soils and from the EMMV‐t0 method were 5.7%–13.6% and 20.6% higher than that calculated by the ESV method, respectively. Nevertheless, in the C‐poor lands, no significant algorithmic biases of SOC estimation were observed. Finally, both the SOC stock discrepancies (ESV vs. EMMV‐t0) and the proportions of this unaccounted SOC were large and site‐dependent. These results suggest that although the modified methods that referenced to t1 soils could reduce the biases derived from soil volume changes, they may not properly quantify SOC changes due to using unconserved reference systems. The EMMV‐t0 method provides an approach to address the two problems and is potentially useful since it enables SOC comparability and integrating SOC datasets.

## 1 INTRODUCTION

Soil organic carbon (SOC) stock and accumulation rate are critical components of key ecosystem services such as soil formation, soil fertility, and climate regulation (Delgado‐Baquerizo et al., 2017; Fang, Yu, Liu, Hu, & Chapin, 2018; Pan et al., 2011). The two most intrinsic issues, which fundamentally limit our understanding of the direction and magnitude of soil C changes, are the quality of the observations and the use of appropriate algorithms. The obstacles to achieving a standardized protocol for obtaining a reliable dataset in SOC monitoring have been well documented (Conant, Ogle, Paul, & Paustian, 2011; Jandl et al., 2014). However, the algorithm‐derived uncertainties in comparing SOC concentration, stock, and accumulation rate have not been fully addressed, which hamper reliable assessments of the status of C storage in terrestrial ecosystems and their response and feedback to natural and/or anthropogenic disturbances.

The simplest way to determine SOC status is to measure SOC concentration (Lee, Hopmans, Rolston, Baer, & Six, 2009). However, the current unit representing SOC concentration, C mass per unit of soil mass (e.g., g C/kg soil), will result in underestimation of SOC amount in soil samples with higher SOC concentration due to the inappropriate inclusion of the numerator (C mass) within the denominator (soil mass; Figure S1). The most common way of determining SOC status is to measure C stock, expressed as C mass per unit of area at a fixed depth (e.g., g C/m^2^ at h depth). SOC stock is conventionally calculated by multiplying soil bulk density with SOC concentration at a fixed soil depth (Adams, 1973; Brimhall et al., 1992; Delgado‐Baquerizo et al., 2017). This conventional method based on equivalent soil volume (ESV) fails to define the total soil mass since it ignores processes of soil swelling or shrinking during soil development or degradation. Consequently, biases arise when the conventional SOC concentrations or SOC stocks are compared across space or over time (Post and Kwon, 2000; Schuur et al., 2015) (Table 1). For instance, the ESV method will underestimate SOC stocks and SOC accumulation rates in developing forests with expanding soil volume.

**Table 1:** Current methods for SOC comparison and their biases

Methods	Sampling depth	Soil mass	Mineral‐matter mass	Mineral‐matter depth	Estimation bias	Data comparability	References
Comparing SOC concentrations	N/A	N/A	N/A	N/A	Underestimated for soils with greater SOC content	Not comparable at per area or volume basis	Lee et al. (2009)
Comparing C stocks							
(a) Equivalent soil volume (ESV)	Not justified	Nonequivalent	Nonequivalent	Not defined	Underestimated for soils with lower BD	Not comparable at per volume basis	Post and Kwon (2000), Schuur et al. (2015)
(b) Equivalent soil mass (ESM)	Justified case by case	Equivalent	Nonequivalent	Not defined	Underestimated for soils with either greater SOC content or greater true density of mineral‐matter	Not comparable at per volume basis; Not comparable across studies sampling different soil mass	Dalal and Mayer (1986), Ellert and Bettany (1995), Mikhailova et al. (2000), Gifford and Roderick (2003), Lee et al. (2009), Wendt and Hauser (2013)
(c) Equivalent soil mineral‐matter mass (EMMM)	Justified case by case	Nonequivalent	Equivalent	Not defined	Underestimated for soils with a greater true density of mineral‐matter	Not comparable at per volume basis; Not comparable across studies sampling different mineral‐matter mass	Poulton et al. (2003), Tremblay et al. (2006)

To overcome the problem of changing soil volume, some researchers introduced an approach based on equivalent soil mass (ESM) to compare SOC stocks at specific local sites (Dalal & Mayer, 1986; Ellert & Bettany, 1995; Lee et al., 2009; Mikhailova, Bryant, Vassenev, Schwager, & Post, 2000). In the ESM method, soil sampling depths were adjusted so that the same soil mass (SM) could be measured. Furthermore, a simplified ESM method which determining SOC stocks through cumulative mass coordinates without bulk density sampling was developed (Gifford & Roderick, 2003; Wendt & Hauser, 2013). The ESM methods could be further modified as those based on equivalent mineral‐matter mass (EMMM) to minimize the bias in SOC stock comparisons that induced by the difference of soil organic matter (SOM) among samples (Poulton, Pye, Hargreaves, & Jenkinson, 2003; Tremblay, Périé, & Ouimet, 2006). However, the ESM methods have a similar bias in SOC comparison to that resulting from using the inappropriate unit of SOC concentration which including the same variable (SOC) within both the numerator (C mass) and the denominator (soil mass; Table 1). An increase in soil organic matter (SOM) will reduce the sampled amount of soil mineral‐matter (MM); as a result, SOC stocks in samples with greater SOM concentration will be underestimated by the ESM method. Nevertheless, the two mass‐based methods were more appropriate than the conventional ESV method, although they may still underestimate SOC stocks in soils with a higher true density of mineral‐matter (Table 1).

To date, even the EMMM method has not been widely employed. One of the major obstacles limiting the application of the two mass‐based methods may be the low comparability of SOC stocks across space and time. To continue sampling an equivalent soil mass or mineral‐matter mass, a reference soil profile should be chosen so that SOC stocks of other soil samples can be compared. Hence, both the sampled amount of soil mass and the SOC stock changes were sensitive to the conditions of the reference soils (Lee et al., 2009). However, given that samples in fixed soil depth may contain different soil (or soil mineral‐matter) mass, it was difficult to re‐analyze newly and/or previously reported ESV‐based dataset and then re‐assess SOC change patterns using the two mass‐based methods due to the unequal basis for comparison.

**Figure 1:**
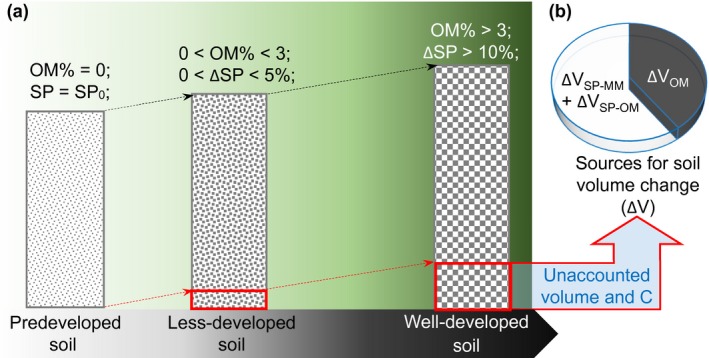
A conceptual framework for the estimation biases of SOC accumulation during forest development. Panel a shows how the unaccounted soil volume and C increase with changes in soil organic matter (SOM) content and soil porosity (SP). Vegetation and soil development statuses are indicated by the gradation of green and black, respectively. The distance between the black and red arrows refers to the soil sampling depth by the conventional approach. Panel b shows the sources of soil volume change: changes in SOM and SP. SP_0_: SP in reference soil; ΔSP: change of SP relative to SP_0_, which consist of the increased SP‐occupied volume within soil mineral‐matter (SP‐MM) and soil organic matter (SP‐OM); Δ*V*_OM_: the true volume of SOM (excluding SP volume within SOM)

We considered that the inherent limitation of either the ESV‐based or the two mass‐based methods is to using reference soil profiles with varied status. To solve this problem, a conserved reference system is needed. We thus introduced a basal mineral‐matter profile, which ideally contains no organic matter but has a natural basal porosity that can be approximated with the mineral soil in deeper layers, as the true time zero reference system. Given that soil mineral‐matter characteristics (true density and natural porosity) may differ with soil types and climate, a fixed volume of basal mineral‐matter rather than a fixed mass of basal mineral‐matter is an ideal reference soil profile. Importantly, the unaccounted volume of the basal mineral‐matter could be quantified by changes in SOM content and soil porosity (SP). We partitioned the soil into three components: mineral‐matter (MM), organic matter (OM), and soil porosity (SP). Hence, the process of soil formation can be described as increases in OM content and SP from a base volume of MM with original porosity. Then, a volume of newly increased OM and/or SP will exclude a volume of MM resulting in a miscount of the associated SOC by the ESV method (Figure 1a). The unaccounted volume of basal MM for a given soil sample is equal to the soil volume change (ΔV) which can be calculated by relative changes in OM and SP status compared to those in the basal MM profile (Figure 1b). Thereby, any given soil profile with fixed sampling volume (*V*_0_) could be standardized into a new soil profile with new volumes (*V*_0_ + Δ*V*) that contain an equivalent volume of the basal MM. Accordingly, both the SOC stocks and their changes could be estimated and compared based on equivalent volume of basal mineral‐matter (EMMV‐t0).

Furthermore, the introduction of the time zero reference system may improve the accuracy of estimating SOC changes. The estimation bias of SOC change rate in a fixed sampling depth may decline with the increase of the included mass of soil mineral‐matter. Thus, soil profiles at earlier developmental stages are more appropriate to be selected as the reference soil systems. Therefore, we considered that our new approach of EMMV‐t0 would improve both the data comparability and the accuracy of estimating SOC stock at fixed sampling depth.

To show the algorithm‐induced biases in SOC monitoring over time, we compared the SOC change rates calculated by all the methods of ESV, ESM, EMMM, EMMV‐t1, and EMMV‐t0 using two representative datasets derived from relatively C‐rich mature forests and C‐poor afforested lands, respectively (Figure S2). In addition, to show the bias magnitude in SOC stock comparisons across space, the differences of SOC stocks for the whole soil profiles that were calculated by the ESV and EMMV‐t0 methods were examined using datasets from six distinct sites across three biomes.

## 2 MATERIALS AND METHODS

### 2.1 The conventional method based on equivalent soil volume (ESV)

The conventional method can be expressed as:

(1) $${\text{ESV - C}}_{\text{stock}}=0.50/1000\times \sum_{i=1}^{n}\left({\text{BD}}_{i}\times h_{i}\times 10000\times {\text{OM}}_{i}\right)$$

where ESV‐C_stock_ is SOC stock calculated by the conventional ESV method (g C/m^2 ^in fixed sampling soil volume), 0.50 is the conversion factor from OM to C (Pribyl, 2010), 1,000 is the conversion factor from per gram of soil mass to per kilogram of soil mass, “*i*” refers to a given sampled soil layer, BD is soil bulk density (g/cm^3^), h is sampling depth (cm), 10,000 is the conversion factor from a given sampling area (cm^2^) to one square meter, and OM is SOM concentration (g OM/kg soil).

### 2.2 The method based on equivalent soil mass (ESM)

The ESM method can be expressed as:

(2) $${\text{ESM - C}}_{\text{stock}}={\text{ESV - C}}_{\text{stock}}+\text{ESM -}\;\Delta C_{\text{stock}}$$

(3) $$\text{ESM}-\Delta C_{\text{stock}}=0.50\times \Delta \text{SM}\times {\text{OM}}_{n+1}$$

where ESM‐C_stock_ is SOC stock calculated by the ESM method (g C/m^2 ^in varied sampling soil volume with fixed mass of soil), ESM‐ΔC_stock _is SOC stock (g C/m^2^) in the additional sampled soil, ΔSM is the amount of soil mass (kg soil/m^2^) that should be additionally sampled to obtain an equivalent soil mass, and OM*_n_*_ + 1 _is the organic matter concentration in the additionally sampled layer (“*n + *1” layer) of soil (g OM/kg soil).

### 2.3 The method based on equivalent mineral‐matter mass (EMMM)

The EMMM method can be expressed as:

(4) $${\text{EMMM - C}}_{\text{stock}}={\text{ESV - C}}_{\text{stock}}+\text{EMMM -}\;\Delta C_{\text{stock}}$$

(5) $${\text{EMMM - C}}_{\text{stock}}=0.50\times \Delta \text{MM}\times {\text{OMm}}_{n+1}$$

where EMMM‐C_stock_ is SOC stock calculated by the EMMM method (g C/m^2 ^in varied sampling soil volume with fixed mass of mineral‐matter), EMMM‐ΔC_stock _(g C/m^2^) is SOC stock in the additional sampled mineral‐matter, ΔMM is the amount of mineral‐matter mass (kg MM/m^2^) that should be additionally sampled to obtain an equivalent mineral‐matter mass, and OMm*_n_*_ + 1 _is the organic matter concentration per unit of soil mineral‐matter (g OM/kg MM) in the additionally sampled layer (“*n + *1” layer), which is equal to OM*_n + _*_1_/(1‐OM*_n + _*_1_/1,000).

### 2.4 The method based on equivalent mineral‐matter volume (EMMV)

The EMMV method can be expressed as:

(6) $${\text{EMMV - C}}_{\text{stock}}={\text{ESV - C}}_{\text{stock}}+\text{EMMV -}\;\Delta C_{\text{stock}}$$

(7) $$\text{EMMV -}\;\Delta C_{\text{stock}}=0.50/1000\times {\text{BDm}}_{n+1}\times \sum_{i=1}^{n}\Delta V_{i}\times {\text{OMm}}_{n+1}$$

where EMMV‐C_stock_ is SOC stock calculated by the EMMV method (g C/m^2 ^in varied sampling soil volume with equivalent volume of basal mineral‐matter), EMMV‐ΔC_stock _is SOC stock (g C/m^2^) in the additional sampled volume of mineral‐matter, BDm*_n_*_ + 1_ is the bulk density of soil mineral‐matter (g MM/cm^3^ soil) in the additionally sampled layer (“*n + *1” layer), which is equal to (BD*_n + _*_1_ − BD*_n + _*_1_ × OM*_n + _*_1_/1,000), and ΔV_i_ refers to the volume change (Δ*V*) in the “*i*”th layer of the soil profile (cm^3^/m^2^). The details for estimating the Δ*V* are shown separately below.

If the basal mineral‐matter reference systems (soils at time zero with natural porosity but no organic matter) were used, the EMMV method was named EMMV‐t0; if reference soils at specific developmental stages (e.g. at t1, soils at the beginning of a given study) were used, the EMMV method was named EMMV‐t1. Note that the most distinct feature of the EMMV‐t0 method relative to all previous methods is quantifying SOC stocks with reference to the basal mineral‐matter profiles. To show the potential influences of the choice of reference soils on the bias of estimating SOC changes, we also compared the SOC change rates calculated by the EMMV‐t0 method with that calculated by the EMMV‐t1 method.

### 2.5 Estimation of soil volume change

The volume increases in OM (Δ*V*_OM_) and SP (Δ*V*_SP_) are two major sources of soil volume change (Δ*V*). They can be estimated by comparing OM and SP in the basal reference mineral‐matter (OM = 0; SP = SP_0_) with those in the studied soils. The main equations are given below:

(8) $$\Delta V=\sum_{i=1}^{n}\left(\Delta V_{\text{OM}i}+\;\Delta V_{\text{SP}i}\right)$$

(9) $$\Delta V_{\text{OM}i}=V_{\text{OM}i}$$

If the soil profile contains several layers (*n* > 1), *V*_OM_ and Δ*V*_SP_ in the “*i*”th layer (*i* ≤ *n* − 1) can be calculated as:

(10) $$V_{\text{OM}i}={\text{BDm}}_{i}\times \left(V_{i}/1000\right)\times \left({\text{OMm}}_{i}/1.3\right)$$

(11) $$\Delta V_{\text{SP}i}=\Delta {\text{SP}}_{i}\times V_{i}$$

Thus,

(12) $$\Delta V_{i}=\left({\text{BDm}}_{i}\times \left(V_{i}/1000\right)\times \left({\text{OMm}}_{i}/1.3\right)\right)+\left(\Delta {\text{SP}}_{i}\times V_{i}\right)$$

For the deepest soil layer (*i* = *n* and *n* > 1) or if the soil profile contains only one layer (*n* = 1), *V*_OM_ and Δ*V*_SP_ in the “*n*” layer can be calculated as:

(13) $$V_{\text{OM}n}={\text{BDm}}_{n}\times \left(\left(V_{n}+\Delta V_{n}\right)/1000\right)\times \left({\text{OMm}}_{n}/1.3\right)$$

(14) $$\Delta V_{\text{SP}n}=\Delta {\text{SP}}_{n}\times \left(V_{n}+\Delta V_{n}\right)$$

Thus,

(15) $$\Delta V_{n}=\left(\left({\text{BDm}}_{n}/1000\right)\times \left({\text{OMm}}_{n}/1.3\right)+\Delta {\text{SP}}_{n}\right)/\left(1-\left(\left({\text{BDm}}_{n}/1000\right)\times \left({\text{OMm}}_{n}/1.3\right)+\Delta {\text{SP}}_{n}\right)\right)\times V_{n}$$

Here,

(16) $${\text{BD}}_{m_{i}}={\text{BD}}_{i}-{\text{BD}}_{i}\times {\text{OM}}_{i}/1000$$

(17) $${\text{OMm}}_{i}={\text{OM}}_{i}/\left(1-{\text{OM}}_{i}/1000\right)$$

(18) $$\Delta {\text{SP}}_{i}={\text{SP}}_{i}-{\text{SP}}_{0}$$

where 1.3 is the average true density of OM (g/cm^3^) which excluding the influence of porosity within OM (Adams, 1973; Hillel, 2004); SP_0_ refers to the soil porosity in the time zero reference soil.

### 2.6 Algorithm‐induced biases in estimating SOC change rates

We compared SOC change rates recalculated by the conventional soil volume‐based method (ESV), the two mass‐based methods (ESM and EMMM), and the mineral‐matter volume‐based approach (EMMV‐t0 and EMMV‐t1) using datasets from the mature tropical forests of the Dinghushan Mountains, China (Zhou et al., 2006) and from afforested agricultural lands with different stand ages in northeastern China (Mao, Zeng, Hu, Li, & Yang, 2010). The two sites represent C‐rich mature natural lands and C‐poor restoring degraded lands, respectively.

#### 2.6.1 SOC accumulation rate in the mature tropical forest of the Dinghushan Mountains

To quantify the algorithm‐derived biases in estimating SOC accumulation rate, we re‐analyzed the soil C dataset from the mature monsoon evergreen forests at Dinghushan Mountain where soil C had been monitored over 20 years (Zhou et al., 2006). We used the two equations (SOC = 0.035 × Years–67.97, *R* ^2^ = 0.90, *p* < 0.0001 and BD = −0.0032 × Years + 7.42, *R* ^2^ = 0.90, *p* = 0.01) (Zhou et al., 2006) to calculate the SOC (%) and BD in topsoil (0–20 cm) from 1979 to 2003. The SOC concentrations in the additional sampled soils (OM*_n_*_ + 1 _or OMm*_n_*_ + 1_) were approximately half of those found in the 0–20 cm soil layer for each year (Figure S2a). The BD_m_, OM_m_, ΔSP, and Δ*V* for the topsoil (0–20 cm) were calculated by the Equations ((13), (14), (15), (16), (17), (18), (19), (20), (21), (22), (23)); given that the BD_m_ in the additional sampled soils (“*n + *1” layer, 20–40 cm) were not directly measured, it was assumed to be equivalent to that of the 0–20 cm layer to obtain conservative estimations of the unaccounted amount of soil mineral‐matter by the EMMV method. In addition, a zero‐time porosity of 41.8% was used, which was approximated with the average soil porosity in deep layers >40 cm (Table S1). The SOC stock in the top soils (0–20 cm and additional included depth of soil) for a given year (C_stock‐year_) was calculated by each of the five methods. Accordingly, the SOC stock change rates (*K*C_stock_, g C/m^2^ year) were calculated as:

(19) $$KC_{\text{stock}}=\text{Slope}\left(C_{\text{stock - t1}}:C_{\text{stock - t2}}\right)$$

where t1 and t2 refer to the beginning (1979) and end (2003) of the SOC monitoring study in the Dinghushan Mountains, respectively.

#### 2.6.2 SOC accumulation rate in afforested agricultural lands in northeastern China

The SOC stock change rates were calculated by all the five methods to show the algorithm‐derived biases in lands with relatively C‐poor conditions. The dataset was from a study employing a “space‐for‐time” substitution approach (Mao et al., 2010). SOC stock status in afforested lands with a stand age of 0 (control agricultural system), 5, 10, 15, and 20 years was estimated by all the five methods. Hence, both the SOC stock loss rates during years 0–10 and the SOC accumulation rates during years 10–20 in topsoil (0–15 cm) were calculated using Equation (19). The zero‐time porosities were approximated with the average soil porosity in deep layers of 60–100 cm. The SOC concentrations in the additional sampled soils (OM*_n + _*_1_ or OMm*_n + _*_1_) were approximated by directly measured SOC concentrations in the 15–30 cm soils. The BD_m_, OM_m_, ΔSP, and ΔV for the topsoil (0–15 cm) were calculated by the Equations (13)–(23); the BD_m_ in the additional sampled soils (“*n + *1” layers) were also approximated by directly measured SOC concentrations in the 15–30 cm soils.

### 2.7 The comparability of SOC stocks at six sites across biomes

SOC stocks at six sites from boreal, temperate, and tropical regions (Guo & Wang, 2014; He et al., 2013; Hu & Liu, 2013; Shi et al., 2012; Wang, Zhao, Xu, Wang, & Peng, 2013; Xin, Zou, & Zhao, 2014) were calculated with both the ESV and EMMV‐t0 methods to show the magnitude of low comparability that the conventional ESV method may have induced. Two sites with distinct characteristics were selected for a given biome to obtain general results. The unaccounted SOC stocks, indicated by the discrepancy between SOC stocks calculated by the ESV method and the EMMV‐t0 method, were calculated throughout the whole profile at varying depths (5–40 cm). The BD_m_, OM_m_, ΔSP, and ΔV for soil profiles at a given depth were calculated by the Equations (13), (14), (15), (16), (17), (18), (19), (20), (21), (22), (23). For each plot (one site may have several plots), BDm*_n + _*_1_ and OMm*_n + _*_1_ were calculated with those directly measured values of BD*_n + _*_1_ and OM*_n + _*_1_ in the “*n + *1” layer for a given depth of soil profile. For example, the data from soil layer of 10–20 cm were used as data of the “*n + *1” layer when SOC stock in a top soil profile (0–10 cm) from the boreal site 1 was calculated. Afterward, the unaccounted SOC for each soil profile, plot, and site were calculated by Equation (7), and then, the proportions of unaccounted SOC relative to that calculated by the ESV method could be calculated.

### 2.8 Statistical analysis

One‐way ANOVA was performed to show the algorithmic influences on the SOC change rates in afforested lands with increasing stand ages and to show the site effects on the magnitude of algorithm‐derived biases (unaccounted SOC stocks when comparing ESV‐calculated SOC stocks with EMMV‐t0 calculated SOC stocks, and their proportions relative to the ESV‐calculated SOC stocks) in the 0–30 cm soil profiles. All statistics were performed with SPSS 19.0 (IBM).

## 3 RESULTS

### 3.1 Algorithm‐derived biases in estimating SOC change rates

**Figure 2:**
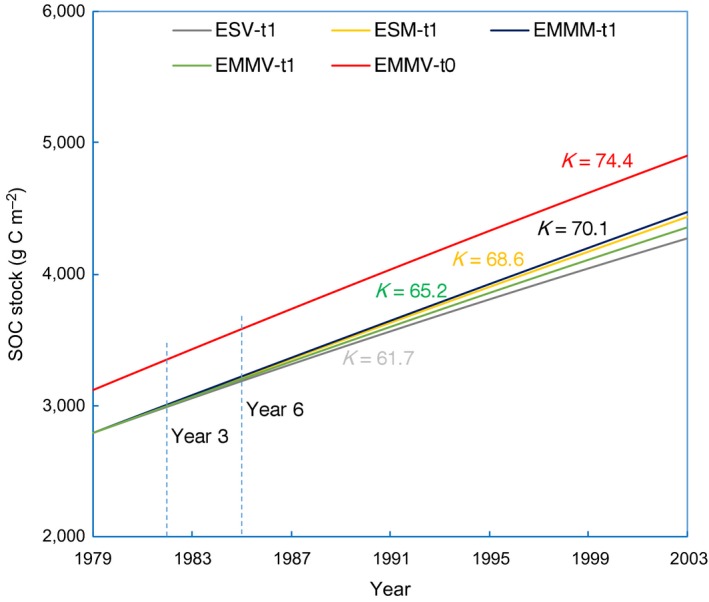
Re‐estimated annual SOC accumulation rates in a mature forest. The SOC accumulation rates, represented as linear slopes (*K*), were recalculated by both the previously used methods and our modified methods using a dataset from the mature tropical forests in the Dinghushan Mountains (Zhou et al., 2006). ESV: the conventional equivalent soil volume method; ESM: the equivalent soil mass method; EMMM: the equivalent mineral‐matter mass method; EMMV: the equivalent mineral‐matter volume method. The “t0” and “t1” indicates using the basal mineral‐matter profile at time zero and the soil profile at time t1 (1979, the year that soil monitoring was initiated) as reference soils for the estimation of SOC change, respectively. The dotted lines indicate the years at which notable algorithm‐induced biases of SOC accumulation rate may occur

The SOC accumulation rates in the topsoil (0–20 cm) of mature tropical forests in the Dinghushan Mountains calculated by the five different methods can be separated into three distinct groups (Figure 2). The conventional ESV method and the new EMMV‐t0 approach obtained the lowest (61.7 g C/m^2^ year) and highest (74.4 g C/m^2^ year) SOC accumulation rate, respectively, suggesting that, on average, 20.6% of the annual accumulated SOC may be underestimated by the conventional ESV method. The two mass‐based methods of ESM and EMMM and the EMMV‐t1 method (using soil profiles from 1979 as reference systems) obtained intermediate levels of SOC accumulation rates of 68.6, 70.1, and 65.2 g C/m^2^ year, which was 11.2%, 13.6%, and 5.7% greater than that calculated by the ESV method, respectively. In addition, the algorithm‐induced biases in SOC change estimations were not noticeable during the first 6 years when comparing between the three modified methods that referenced to soils at time t1 (ESM, EMMM, and EMMV‐t1), the modified method that referenced to soils at time zero (EMMV‐t0), and the conventional ESV method.

**Figure 3:**
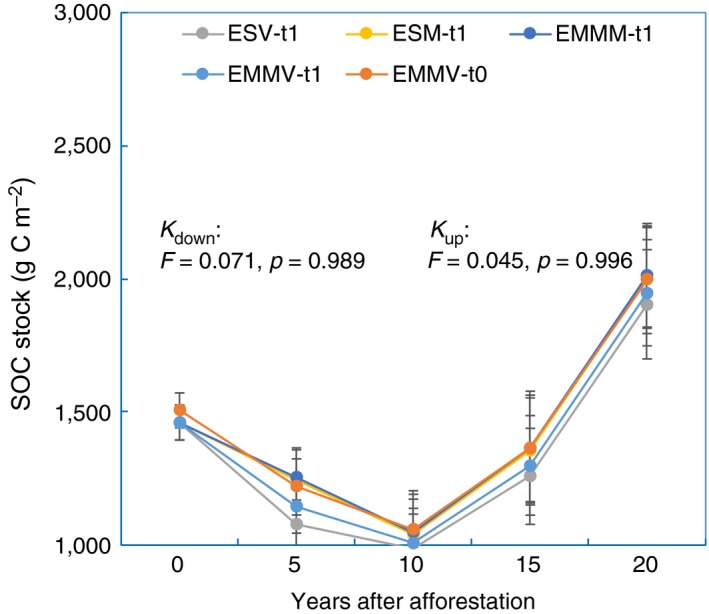
Re‐estimated annual SOC change rates in afforested agricultural lands. The SOC accumulation rates, represented as linear slopes (*K*), were recalculated by both the previously used methods and our modified methods using a dataset from afforested agricultural lands with different stand ages in northeastern China (Mao et al., 2010). ESV: the conventional equivalent soil volume method; ESM: the equivalent soil mass method; EMMM: the equivalent mineral‐matter mass method; EMMV: the equivalent mineral‐matter volume method. The “t0” and “t1” indicates using the basal mineral‐matter profile at time zero and the soil profile at time t1 (the nonafforested agricultural lands) as reference soils for the estimation of SOC change, respectively. Error bars represent standard errors (*n* = 3)

In contrast, in the C‐poor afforested agricultural lands in northeastern China, no significant algorithm‐induced biases of either SOC loss rates (*K*_down_, *F* = 0.071, *p* = 0.989) or SOC accumulation rates (*K*_up_, *F* = 0.045, *p* = 0.996) in the topsoil (0–15 cm) were observed (Figure 3). Note that the SOM concentrations declined sharply at a depth of 15 cm and were consistently low in the subsurface soil layers (>15 cm) either at year‐zero lands (ranging from 3.4 ± 0.7 to 7.2 ± 0.6 g OM/kg soil) or at year‐20 lands (ranging from 5.8 ± 0.4 to 10.0 ± 1.5 g OM/kg soil; Figure S2b).

### 3.2 Algorithm‐derived biases in comparing SOC stocks across biomes

**Figure 4:**
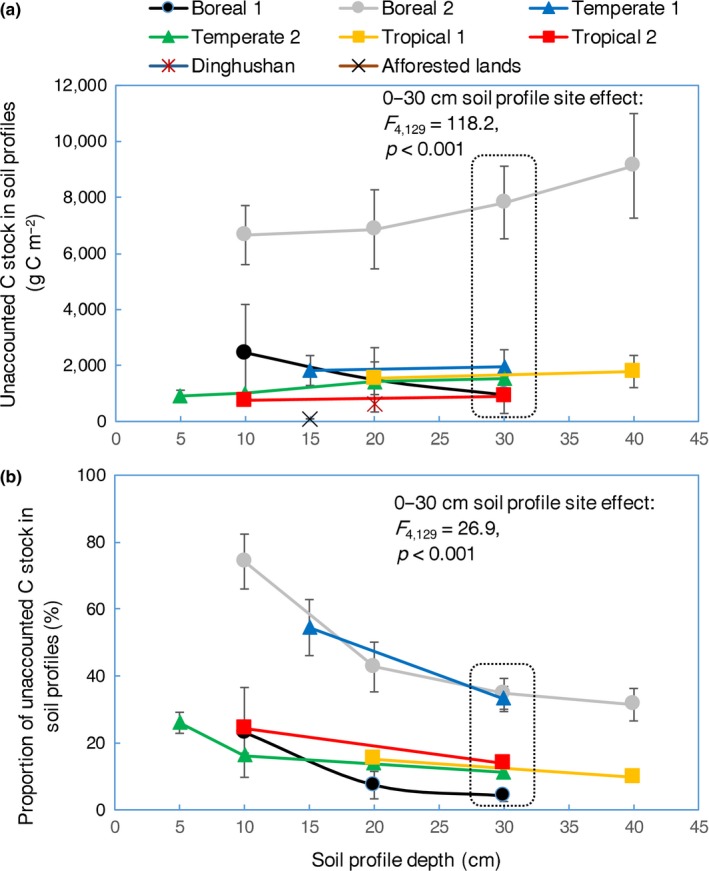
Unaccounted SOC stocks in the whole soil profiles from representative sites across biomes. Mean values are shown ± 1 *SE*; tropical site 1 was not included when performing ANOVA to show the site effect on the amount and proportion of unaccounted SOC (0–30 cm) due to data not available. To indicate the potential biases of estimating SOC change rates that the unaccounted SOC stocks at the six representative sites may accompany with, the unaccounted SOC stocks from the studied mature forests in Dinghushan (0–20 cm, in 2003) and the afforested lands (0–15 cm, based on data in 20‐year‐old afforested lands) were also showed in panel a

The spatial comparability of SOC stocks estimated by the ESV method and EMMV‐t0 method will theoretically be the lowest and highest, respectively. The unaccounted SOC stocks, indicated by the difference between SOC stocks calculated by the ESV method and SOC stocks calculated by the EMMV‐t0 method, represent the magnitude of algorithm‐induced bias. To explore the change patterns of algorithm‐induced biases across biomes, we calculated the unaccounted SOC stocks in soil profiles at varied depths (5–40 cm) from six representative forest sites. The unaccounted forest SOC stocks ranged from 950 ± 665 to 9,127 ± 1,867 g C/m^2^, from 909 ± 206 to 1,952 ± 610 g C/m^2^, and from 755 ± 31 to 1783 ± 575 g C/m^2^ in boreal, temperate, and tropical sites, respectively (Figure 4). If the SOC stocks at the top 30 cm profiles were focused, the unaccounted SOC stocks by the ESV method, which ranged from 892.6 ± 37 g C/m^2^ to 7,819.3 ± 1,296 g C/m^2^, differed significantly across the six representative sites (*F*_4,129_ = 118.2, *p* < 0.001) (Figure 4a). Notably, the unaccounted SOC stocks in the aforementioned mature forest soils (0–20 cm) and the 20‐year‐old afforested lands (0–15 cm) were only 629.8 and 93 g C/m^2^, respectively, which were much lower than those from the six sites (Figure 4a). Also, the proportions of unaccounted SOC stocks in this top soil layer, which ranged from 4.18 ± 1.7% to 34.66 ± 4.6%, differed significantly across the sites (*F*_4,129_ = 26.9, *p* < 0.001; Figure 4b).

## 4 DISCUSSION

Although SOC status in a specific soil can be characterized either by SOC concentration (g C/kg soil) or by the ESV‐, ESM‐, or EMMM‐based SOC stocks, large algorithm‐derived biases may occur if SOC status is compared across space or over time. We examined all the previously used methods and revealed that the algorithm‐derived biases in SOC monitoring may be primarily derived from three sources: the inappropriate unit of SOC concentration or stock, the unaccounted soil volume change, and the using of nonconserved reference soil systems. Accordingly, we introduced the conserved reference system of time zero mineral‐matter and thus determined SOC stock as C mass in an equivalent volume of basal mineral‐matter (EMMV‐t0). This alternative approach theoretically overcomes the aforementioned limitations and potentially improves the accuracy of SOC change estimation and comparability of SOC stocks.

Firstly, we observed large algorithm‐induced biases in estimating SOC changes over time in the mature tropical forests of the Dinghushan Mountains (Figure 2). The biases here were primarily derived from the soil volume change and the use of unconserved reference system. As expected, the SOC accumulation rates derived from the two mass‐based methods (ESM and EMMM) were greater than that from the conventional ESV method. However, the differences among SOC accumulation rates derived from the methods of ESM and EMMM were very small, suggesting that the biases resulting from ignoring changes in SOM over time were much limited for soils with an intermediate level of SOM concentration (<50 g OM/kg soil), while the slight lower SOC accumulation rate derived from the EMMV‐t1 method compared with that derived from the two mass‐based methods was unexpected. It may result from our assumption that BD in the “*n + *1” layer of soils (20–40 cm) being equal with that in the “*n*” layer of soils (0–20 cm), which may cause underestimation of the unaccounted mineral‐matter mass and thus the SOC stock change rates by the two EMMV methods. In other words, the SOC stocks calculated by the two mass‐based methods and the EMMV‐t1 method would be more similar if a direct measured BD in the “*n + *1” layer of soils were available.

**Figure 5:**
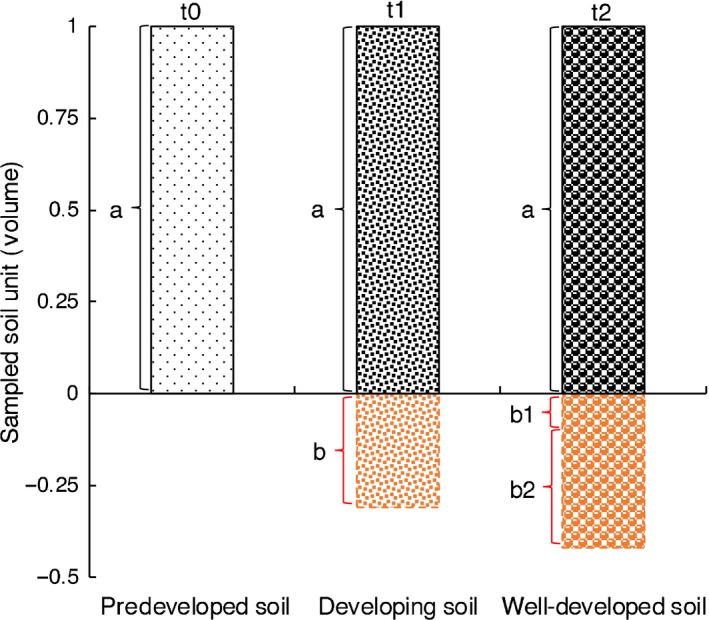
A diagram showing how the selection of reference systems affects the estimation of SOC changes. The three columns at t0, t1, and t2 include different total sampling volume but equivalent volume of basal mineral‐matter. For EMMV‐t0 and EMMV‐t1 method, SOC stock at t2 is compared with that at t0 and t1, respectively. Where “*a*” refers to the fixed sampling volume by the conventional ESV method; “*b*” refers to the additional sampled volume at a given time of t1 to obtain equivalent volume of basal mineral‐matter compared with that at time t0; “*b*1” refers to the additional sampled volume to obtain equivalent volume of basal mineral‐matter compared with that at time t1; “*b*2” refers to the additional sampled volume at time t2 to obtain equivalent volume of basal mineral‐matter compared with that in the soil layer “*b*” at time t1. In other words, the mineral‐matter volume (MMV) in “t1*_a_*” is equal to MMV in “t2*_a_* + t2*_b_*_1_”, MMV in “t1*_b_*” is equal to MMV in “t2*_b_*_2_”, and the MMV in “t0*_a_*” is equal to MMV in “t1*_a_* + t1*_b_*” or MMV in “t2*_a_* + t2*_b_*_1_ + t2*_b_*_2_”

Notably, the selection of reference systems may theoretically exert predominant influence on the estimated SOC change rates. For instance, in developing ecosystems, SOC accumulation rates estimated by the EMMV‐t0 method are theoretically higher than that estimated by the EMMV‐t1 method (using reference soils at specific developmental stages, e.g. soils at the beginning of a given study; Figure 5). The SOC accumulation rate during a given period (Δ*t*, from t1 to t2) (*K*C_stock‐t1_) can be calculated by the EMMV‐t1 method as:

(20) $${\text{KC}}_{\text{stock - t1}}=\left(\left(C_{\text{stock - t2 -}\;a}+C_{\text{stock - t2 -}\;b1}\right)-C_{\text{stock - t1 -}\;a}\right))/\Delta t$$

whereas the SOC accumulation rate during a given period (Δt, from t1 to t2) (*K*C_stock‐t0_) can be calculated by the EMMV‐t0 method as:

(21) $${\text{KC}}_{\text{stock - t0}}=\left(\left(C_{\text{stock - t2 -}\;a}+C_{\text{stock - t2 -}\;b1}+C_{\text{stock - t2 -}\;b2}\right)-\left(C_{\text{stock - t1 -}\;a}+C_{\text{stock - t1 -}\;b}\right)\right)/\Delta t$$

As a result,

(22) $$KC_{\text{stock - t0}}=KC_{\text{stock - t1}}+\left(C_{\text{stock - t2 -}\;b2}+C_{\text{stock - t1 -}\;b}\right)/\Delta t$$

where “a” refers to sampled soil volume by the ESV method; “b” refers to the additional volume of soil that should be resampled at a given time of t1 to obtain equivalent volume of basal MM compared with that at time t0; “b1” refers to the additional volume of soil that should be resampled to obtain equivalent volume of basal MM compared with that at time t1; “b2” refers to the additional volume of soil that should be resampled at time t2 to obtain equivalent volume of basal MM compared with that in the soil layer of “b” at time t1.

Given that the SOC densities at later stages (t2) are greater than that at earlier stages (t1) for a developing soil system, we conclude that

(23) $$C_{\text{stock}-\text{t2}-b2}-{\;C}_{\text{stock}-\text{t1}-b}>0,$$

and thus,

(24) $$KC_{\text{stock - t}0}>KC_{\text{stock - t1}}$$

This theoretical inference was supported by the large discrepancy of SOC accumulation rates in the mature forests in Dinghushan Mountains that calculated by the methods of EMMV‐t0 and EMMV‐t1. EMMV‐t0 method, which used conserved basal mineral‐matter profiles as reference systems, obtained greater SOC accumulation rate than that derived from the EMMV‐t1 method. In fact, the EMMV‐t0 method and EMMV‐t1 method accounted for SOC stocks in 20 and 15.3 cm of the basal mineral‐matter for forest soils in the Dinghushan Mountains, respectively. The EMMV‐t1 method did not fully reflect the unignorable subsurface (20–30 cm) SOC incorporation (Figure S2a). Thus, we observed 14.1% excess annual SOC accumulation in Dinghushan forests when estimated by the EMMV‐t0 method compared with that estimated by the EMMV‐t1 method (Figure 2). Nevertheless, our results also showed that the algorithm‐biases in the mature forests of Dinghushan Mountains were almost not detectable during the first 6 years of SOC monitoring, implying that long‐term studies are the most critical in estimating SOC change patterns. On the other hand, the nonsignificant algorithm‐induced biases in estimating SOC changes in the C‐poor afforested lands (Mao et al., 2010) were also understandable because the sampled topsoil (0–15 cm) had accounted for most of the SOC input and soil volume change (Figure 3; Figure S2b). Therefore, it is not suitable to use nonconserved soil profiles, for example, soils at a specific developmental stage (t1, e.g., at the beginning of a specific study), as reference systems for SOC monitoring except for extremely SOC‐depleted ecosystems. However, this is exactly what previously reported methods have done. Such biases in SOC comparisons derived from using unsuitable reference soils have not been recognized in previous studies.

Secondly, the algorithm‐derived biases in comparing SOC stocks across space were also prevalent, large, and inconsistent. The discrepancy (unaccounted C) between the ESV‐calculated SOC stocks and the EMMV‐t0 calculated SOC stocks reflected the bias magnitude in comparing SOC stocks across space. The spatial comparability of ESV‐estimated SOC stocks may be high if the discrepancy was small or consistent across space. However, we found that the discrepancies of SOC stocks were large and site‐dependent. The highest unaccounted C can be eight times greater than the lowest unaccounted C in the boreal forest soils and two times greater in the temperate or tropical forest soils (Figure 4). Note that the unaccounted C in the mature forests in Dinghushan Mountains was much lower than those in the six sites across biomes (Figure 4a). This may imply that the biases in estimating SOC change rates are likely to be larger than we observed in the case of Dinghushan Mountains. Furthermore, the inconsistent proportion of unaccounted C across biomes suggested that the bias magnitudes in estimating SOC change rate by the ESV method may also differ significantly across the six sites. Therefore, the spatial comparability of both the ESV‐estimated SOC stocks and change rates were low in the six representative sites across biomes.

Collectively, our results suggest that the algorithm‐derived biases in quantifying SOC changes can be attributed to not only ignoring soil volume changes but also using unconserved reference soils. Given that mineral weathering may change the true density of soil mineral‐matter (Riebe, Kirchner, Granger, & Finkel, 2001; White et al., 1996), the two mass‐based methods (ESM and EMMM) could partly reduce the biases in SOC estimation derived from soil volume changes, but at the same time will induce new biases in comparing SOC changes among soils with various true density of mineral‐matter. The mineral‐matter volume‐based method with unconserved reference soils (EMMV‐t1) could reduce the biases in SOC estimation either derived from soil volume changes or derived from the varied true density of mineral‐matter. However, all the three methods may not fully account for SOC changes due to using unconserved reference systems, especially in C‐rich ecosystems. The inappropriate use of reference systems may reduce data comparability of SOC stocks across space, reduce data comparability of SOC stock change rates across studies (same or different study sites), and reduce the estimation accuracy of SOC change rates at a given site. The EMMV‐t0 method, which is the unique method that referenced to the basal soils at time zero, provides an alternative approach to address the two major algorithmic problems and thus enables SOC comparability across space and time.

To fully quantify the SOC changes, the ideal situation is to obtain SOC and BD data of the entire soil profile down to the soil–rock interface, or at least to a deeper depth such as 1 m. However, it was too expensive and time‐consuming to be widely applied or rely heavily on modeling and simulation. The EMMV‐t0 approach provides an alternative efficient way to revisit conventional direct measurement datasets (Lu et al., 2018; Minasny et al., 2017; Stockmann et al., 2013) and re‐assess SOC stock change patterns in global top soils.

The major obstacles for the EMMV‐t0 method are to obtain high‐quality datasets of the basal SP in time zero mineral‐matter (SP0) and of the soil volume change. The EMMV‐t0 method can be performed in three steps: (a) characterizing the reference system of time zero mineral‐matter, (b) calculating the volume change in the targeted volume of soils, and (c) measuring SOC concentration in the additional sampled soil layers. We recommend to first measure SP in several soil samples from deeper layers (e.g., >100 cm) and use the average value as an approximation of the basal SP in time zero mineral‐matter (SP0) for a specific site. Second, sample the top layers of soils (e.g., 0–30 cm) and the next deeper soil layer (<10 cm); thereby, both the SOC stocks in topsoil and the SOC concentrations in the additional sampled soil layers can be measured. Finally, SOC stocks at a fixed depth in topsoil (e.g., 30 cm) could be standardized into SOC stocks at various soil depths (30 cm plus Δ*h*, the additional depths, cm), which includes an equivalent volume of basal mineral‐matter. In case of nonuniform soils, it is better to sample the soil profile by its natural layers and then measure the soil porosity and organic matter content in each layer. But it is very time‐consuming and we need to make the decision according to the trade‐off between data precision and practicability. Overall, this study explores the bias sources in SOC quantifying and comparisons and establishes an alternative algorithm to improve monitoring of SOC status changes. This new approach may contribute to further studies of measuring precise SOC accumulation for global biogeochemical cycles and for estimating climate change impacts.

## CONFLICT OF INTEREST

The authors declare no conflicts of interests.

## AUTHOR CONTRIBUTIONS

S.F., X.Z., and W.Z. initiated the collaborative study, and W.Z., Y.C., L.S., X.W., S.P., Y.W.L., Y.B.L., R.M., and X.R. contributed to data collecting and compilation, and constructed the database. W.Z., X.L., Y.S., C.C.Z., and S.L. carried out data analyses. W.Z., X.Z., S.F., S.P., and W‐X. Z. wrote the manuscript.

## Data Availability

The datasets are available for download in dryad (DOI: https://doi.org/10.5061/dryad.7465c1j).
