# Supplementary material for: An alternative approach to reduce algorithm‐derived biases in monitoring soil organic carbon changes
Source: Ecol Evol. 2019 May 30;9(13):7586–96. doi: 10.1002/ece3.5308 (PMC6636197; doi:10.1002/ece3.5308)
Supplement: Supplementary file 1 [file ECE3-9-7586-s001.docx]

**Supplementary material for**

An alternative approach to reduce algorithm-derived biases in monitoring soil organic carbon changes

Weixin Zhang^a,b^, Yuanqi Chen^c,j^, Leilei Shi^a^, Xiaoli Wang^d^, Yongwen Liu^e^, Rong Mao^f^, Xingquan Rao^b^, Yongbiao Lin^b^, Yuanhu Shao^a,b^, Xiaobo Li^b^, Cancan Zhao^k^, Shengjie Liu^g^, Shilong Piao^e^, Weixing Zhu^h^, Xiaoming Zou^i,^*, Shenglei Fu,^a,b,^*

* Corresponding authors.

E-mail address: [sfu@scbg.ac.cn](mailto:sfu@scbg.ac.cn) (S. Fu), [xzou2011@gmail.com](mailto:xzou2011@gmail.com) (X. Zou).

This file includes:

**Figure** S1-S2

**Table** S1

**Supplementary References.**


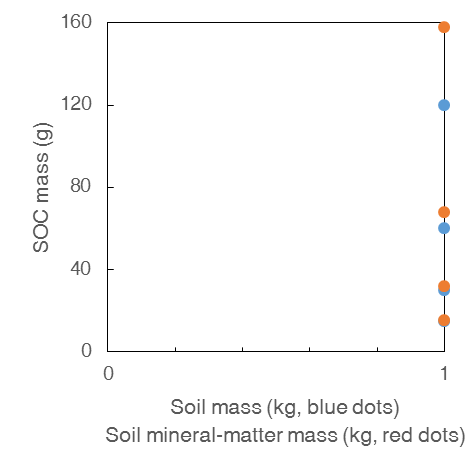


Soil 1

Soil 2

Soil 3

Soil 4

**Figure S1.** **SOC comparison** **biases derived from the current unit of SOC concentration.** Four soils with 15, 30, 60 and 120 g C kg^-1^ soil were used to show how the biases of SOC concentration comparison can be amplified with the increase of SOM mass. The slopes of blue lines and red lines refer to SOC concentrations indicated by g C kg^-1^ soil and g C kg^-1^ mineral-matter, respectively. Note that the distances between the blue and red lines became larger when SOM mass increased.


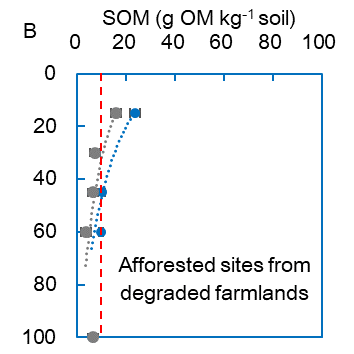

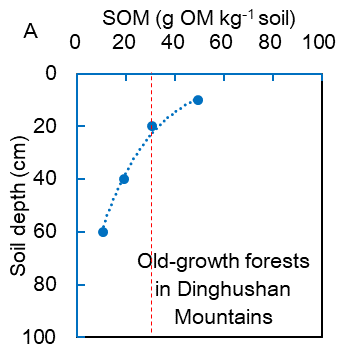


**Figure S2. The SOM distribution pattern according to soil depth in the old-growth forests of the Dinghushan Mountains (A) and in the restoring afforested lands (B)**. Here, data at a given depth (e.g., 20 cm) refers to SOM concentration at a depth interval (e.g., 0 - 20 cm). The data of panel **A** shows the mean values of SOM concentration (error bar data not available) derived from previous study (Fang et al., 2004), and the SOM concentration at a depth of 60 cm was approximated with that at depth of > 40 cm in the original literature. In panel **B**, the gray and blue circles refer to data from the year-zero lands (the control agricultural lands) and year-20 lands (afforested lands with 20-year stand ages), respectively; the error bars are smaller than the size of data points.

**Table S1.** The basal soil porosity (SP0) of the approximated zero-time reference profiles for the studied C-rich and C-poor lands and the six representative sites across biomes. ^†^Calculated with the maximum BD value (1.54 g cm^-3^) in deep soil layers (Fang et al., 2003).

| Site | SP0 (%) | Soil depth (cm) | Soil type | Vegetation | References |
| --- | --- | --- | --- | --- | --- |
| C-rich forests in Dinghushan Mountains | 42.0^†^ | 40-100 | Latosol | Degraded pine forests adjacent to the typical evergreen forests | Fang et al., 2003; Zhou et al., 2006 |
| C-poor afforested lands | 47.19 | 60-100 | Haplic Luvisol | Degraded agricultural lands | Mao et al., 2010 |
| Boreal site 1 | 36.0 | 30-40 | Alfisol, brown coniferous forest soil | Natural secondary *Betula platyphylla* forest | Xin et al., 2014 |
| Boreal site 2 | 30.9 | 40-50 | Gray cinnamon soil | Subalpine meadow | Wang et al., 2013 |
| Temperate site 1 | 26.4 | 40-60 | Typic cinnamon soil | *Pinus tabulaeformis* plantation | Bao and Bao, 2004; Hu and Liu, 2013 |
| Temperate site 2 | 36.7 | 30-40 | Ferralsols, red earth | *Cunninghamia lanceolata* plantation | Guo and Wang, 2014 |
| Tropical site 1 | 34.8 | 40-60 | Ferralsols, red earth | *Cunninghamia lanceolata* plantation | He et al., 2013 |
| Tropical site 2 | 31.3 | 30-60 | Ferralsols, yellow earth | Tropical montane rainforest | Shi et al., 2012 |

**Supplementary references**

Bao, W., & Bao, W. K. (2004). Soil physical and chemical properties under secondary shrub and artificial Chinese pine forest in middle-mountain areas of upper reaches of the Minjiang River. *Bulletin of Soil and Water Conservation*, 24, 10-13. (In Chinese with English abstract)

Fang, Y. T., Mo, J. M., Brown, S., Zhou, G. Y., Zhang, Q. M., & Li, D. J. (2004). Storage and distribution of soil organic carbon in Dinghushan Biosphere Reserve. *Acta Ecologica Sinica*, 24, 135-142. (In Chinese with English abstract)

Fang, Y. T., Mo, J. M., Peng, S. L., & Li, D. J. (2003). Role of forest succession on carbon sequestration of forest ecosystems in lower subtropical China. *Acta Ecologica Sinica*, 23, 1685-1694. (In Chinese with English abstract)
